# Perovskite/Silicon Tandem Solar Cells: From Detailed Balance Limit Calculations to Photon Management

**DOI:** 10.1007/s40820-019-0287-8

**Published:** 2019-07-16

**Authors:** Mohammad I. Hossain, Wayesh Qarony, Sainan Ma, Longhui Zeng, Dietmar Knipp, Yuen Hong Tsang

**Affiliations:** 1 Department of Applied Physics, The Hong Kong Polytechnic University, Hung Hom, Kowloon, Hong Kong People’s Republic of China; 2 Geballe Laboratory for Advanced Materials, Department of Materials Science and Engineering, Stanford University, Stanford, CA 94305 USA

**Keywords:** Perovskite solar cell, Tandem solar cell, Thermodynamic, Photon management, Detailed balance limit

## Abstract

Energy conversion efficiency losses and limits of perovskite/silicon tandem solar cells are investigated by detailed balance calculations and photon management. An extended Shockley–Queisser model is used to identify fundamental loss mechanisms and link the losses to the optics of solar cells. Photon management is used to minimize losses and maximize the energy conversion efficiency. The influence of photon management on the solar cell parameters of a perovskite single-junction solar cell and a perovskite/silicon solar cell is discussed in greater details. An optimized solar cell design of a perovskite/silicon tandem solar cell is presented, which allows for the realization of solar cells with energy conversion efficiencies exceeding 32%.

## Highlights

- Thermodynamic and detailed balance calculations are provided to derive guideline for the optimization of perovskite solar cells.
- The influence of photon management on the energy conversion efficiency of perovskite solar cells is discussed.
- An optimized solar cell design is proposed, which allows for realizing perovskite/silicon tandem solar cell with an energy conversion efficiency exceeding 32%.

## Graphical abstract

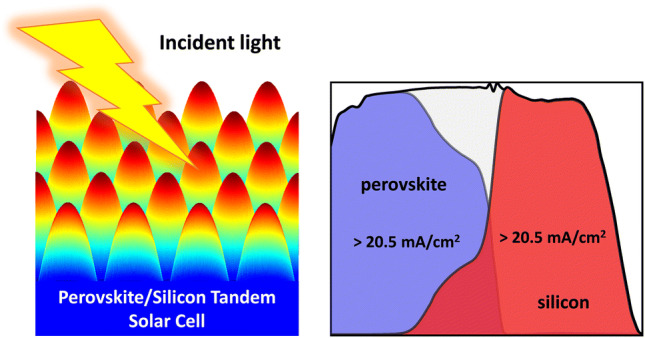

## Introduction

Photovoltaic is the fastest growing energy source in the electricity sector. The cost for production, installation, and maintenance of photovoltaic systems has decreased dramatically throughout the last 10 years. Nevertheless, the technology is not the most widely used primary electrical energy source due to the limited energy conversion efficiency and the system’s cost, which is still high compared to non-renewable energy sources [1–3]. Current commercial solar modules are predominately based on crystalline silicon single-junction solar cells. So far, laboratory solar cells with record energy conversion efficiencies of 26.3% have been demonstrated [4] while the upper theoretical energy conversion efficiency of a solar cell with a bandgap of 1.15 eV (e.g., silicon) is ~ 33.5% [5]. Different approaches have been proposed to increase the energy conversion efficiency of solar cells or to overcome the limits of conventional single-junction solar cells by applying novel physical principles. Based on the proposed approaches multi-junction solar cells have been the most promising approach [6–12]. Detailed balance calculations reveal that serial connected tandem solar cells can reach energy conversion efficiencies exceeding 40% if an ideal material combination is selected for the top and bottom solar cell [5, 6]. Energy conversion efficiencies higher than 40% can be reached if *E*_G_top_ = 0.5 × *E*_G_bot_ + 1.15 eV, where *E*_G_top_ and *E*_G_bot_ are the bandgaps of the top and bottom diode absorbers. The relationship is valid if the bandgap of the bottom diode stays in a range from 0.85 to 1.2 eV. Hence a variety of material combinations can be selected. Crystalline silicon with a bandgap of 1.15 eV is well suited as a bottom solar cell. Hence a lot of research has been devoted to the development of tandem solar cells using a crystalline silicon bottom solar cell. In this case, the highest energy conversion efficiency can be reached if the bandgap of the top cell is equal to ~ 1.7 eV. Several aspects must be considered to combine the well-established crystalline silicon solar cell technology with other material systems or fabrication processes. Amorphous silicon exhibits an ideal bandgap, but the tailstates of the material prevent the realization of solar cells with high open-circuit voltages, which is a prerequisite for the realization of tandem solar cells with high energy conversion efficiencies [13–17]. Silicon oxide/crystalline silicon-based quantum dot and quantum well have been investigated as potential material of the top solar cell [18–21]. However, solar cells with high energy conversion efficiencies have not been realized using silicon-based quantum dots or quantum wells. Furthermore, compound semiconductors have been investigated as potential top solar cell absorber material. However, the high fabrication temperatures of compound semiconductors, the lattice mismatch between silicon and compound semiconductors, and the fabrication cost have so far prevented the successful realization. In recent years, the perovskite material system has been investigated as potential material for single-junction solar cells or as material for perovskite/silicon tandem solar cells [7, 8, 12, 22–24]. So far, the material exhibits very encouraging results [25–32]. High energy conversion efficiencies have been achieved for single-junction solar cells with open-circuit voltages close to the theoretical limit. Furthermore, the material system can be fabricated by a variety of deposition methods at low temperatures, which facilitates the integration of a perovskite top solar cell on a crystalline silicon bottom solar cell. Up to now, perovskite single-junction solar cells with energy conversion efficiencies exceeding 20% have been achieved [33–37]. Research on perovskite/silicon tandem solar cell is still a new research topic. The number of teams working on the realization of record perovskite/silicon tandem solar cells is still small. The realization of perovskite/silicon tandem solar cells with record efficiencies is only possible if the perovskite top solar cell and the silicon bottom solar cell operate very close to the theoretical limit. Nevertheless, perovskite/silicon tandem solar cells with certified energy conversion efficiencies exceeding 27% have been demonstrated [38]. The realization of solar cells with higher energy conversion efficiencies approaching or even exceeding 30% can be expected soon. A thorough investigation of the losses of a solar cell is required to close the gap between theoretical energy conversion efficiency limits and the performance of real solar cells. In this study, we review different thermodynamic and detailed balance approaches used to calculate the upper energy conversion efficiency limit of solar cells. We present several theoretical approaches to determine fundamental energy conversion efficiency limits, starting with the fundamental Carnot process to the well-established Shockley–Queisser limit. However, the Shockley–Queisser limit is still a model making several idealistic assumptions, e.g., the absorber of the solar cell is only described by the bandgap and electrical and optical properties of real materials are not considered. Furthermore, only radiative recombination is considered in the calculation of the upper energy conversion efficiency limit. In Sects. 3.5 and 3.6, we review approaches published by different teams on more generalized detailed balance approaches. Models will be described, which take charge transport processes into account (Sect. 3.5), while optical losses and limits of the absorption of a solar cell, commonly called Yablonovitch limit, are introduced in Sect. 3.6. Section 3 ends with a review of the detailed balance calculations for tandem solar cells. In Sect. 4, we describe how optics and nanophotonics can be used to optimize not only the short-circuit current density of a solar cell, but also all solar cell parameters.

## Fundamentals of Solar Cells

**Fig. 1:**
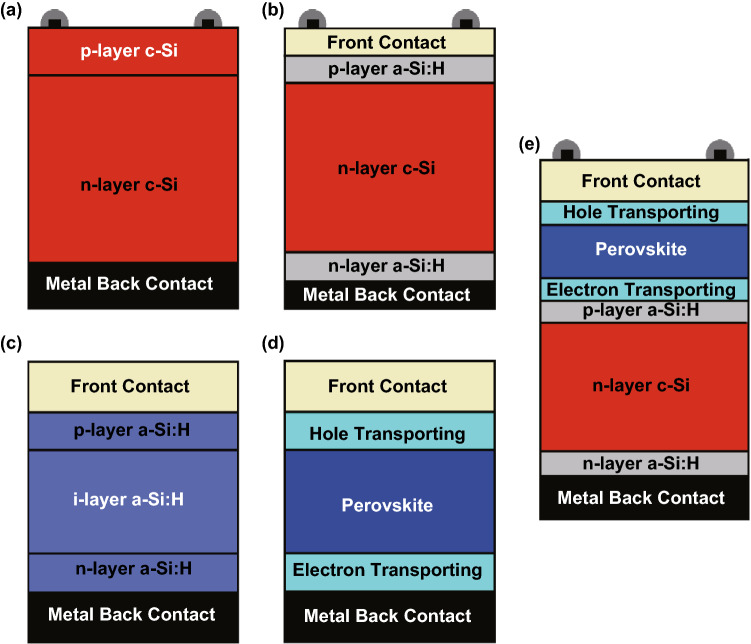
Schematics of **a** crystalline silicon homojunction solar cell, **b** silicon heterojunction solar cell consisting of a crystalline silicon absorber and amorphous silicon contact layers, **c** amorphous silicon homojunction thin-film solar cell, **d** perovskite heterojunction thin-film solar cell, and **e** perovskite/silicon tandem solar cell

In general, a solar cell is an electronic device which converts sunlight into electricity. The basic device structure consists of a p–n or p–i–n junction [3, 39]. In a first step, the incident photons are absorbed causing the creation of electron/hole pairs. In a second step, the photogenerated electron/hole pairs are separated and subsequently collected. The charge collection of the photogenerated charges occurs due to diffusion, drift or the combination of both transport processes to the contacts of the solar cell. Figure 1 provides an overview of different solar cells. Figure 1a shows a schematic sketch of a crystalline silicon homojunction solar cell. Photons are absorbed throughout the complete p–n junction. The photogenerated electron/hole pairs are predominantly collected by charge diffusion. Figure 1b exhibits a heterojunction solar cell consisting of a crystalline silicon absorber and amorphous silicon contact layers. In comparison with a classical silicon homojunction solar cells as shown in Fig. 1a, the heterojunction allows for minimizing optical loss (preferable in the emitter), which leads to a high short-circuit current density. Furthermore, the heterostructure allows for reaching high open-circuit voltages. Typically, amorphous silicon p- and n-layers are used to form the contacts. Due to the high diffusion length of crystalline silicon, charge diffusion is the main charge transport mechanism. Most thin-film solar cells consist of a p–i–n structure. An intrinsic absorber layer is inserted between the p- and n-regions. The charge collection process is mainly or partially dominated by the drift of the electron/hole pairs to the contacts. An example of an amorphous silicon thin-film solar cell is shown in Fig. 1c. A heterojunction thin-film solar cell is shown in Fig. 1d. A perovskite layer is used as an absorber of the incident light. A variety of electron transporting/hole blocking layers and hole transporting/electron blocking layers have been investigated as potential contact layers. In this study, we used transparent conductive oxides (TCO) as contact layers.

In general, the most important parameter to characterize a solar cell is the energy conversion efficiency, which is given by the ratio of the electrical output power density to the optical input power density. An Air Mass 1.5 spectrum is used as a standardized optical input spectrum [40]. The electrical output power density is given by *V*_mp_ × *J*_mp_, where *V*_mp_ and *J*_mp_ are the voltage and current density at the maximal power point. *V*_mp_ and *J*_mp_ are derived from the current–voltage characteristic, *J*(V), of the solar cell. To correlate the *J*(V) characteristics with the physics of a solar cell the parameters, short-circuit current density, fill factor and open-circuit voltage are introduced. The short-circuit current density is given by *J*(*V *= 0) = *J*_sc_, while the open-circuit voltage is determined by *J*(*V* = *V*_OC_) = 0.

1 $$\eta = \frac{{V_{\text{mp}} \times J_{\text{mp}} }}{{P_{\text{in}} }} = \frac{{V_{\text{OC}} \times J_{\text{SC}} \times FF}}{{P_{\text{in}} }}$$

Hence the fill factor can be calculated by Eq. 2:

2 $$FF = \frac{{V_{\text{mp}} \times J_{\text{mp}} }}{{V_{\text{OC}} \times J_{\text{SC}} }}$$

An ideal solar cell can be described by Eq. 3:

3 $$J\left( V \right) = J_{0} \times \left( {\exp \left( {\frac{qV}{{kT_{\text{Cell}} }}} \right) - 1} \right) - J_{\text{SC}}$$

where *q*, *V*, *k*, *T*_Cell_, and *J*_0_ are the elementary charge, applied voltage, Boltzmann constant, the temperature of the solar cell, and the saturation current density. The open-circuit voltage of the solar cell can be determined by Eq. 4:

4 $$V_{OC} = \frac{{kT_{\text{Cell}} }}{q}\ln \left( {\frac{{J_{\text{SC}} }}{{J_{0} }} + 1} \right) \cong \frac{{kT_{\text{Cell}} }}{q}\ln \left( {\frac{{J_{\text{SC}} }}{{J_{0} }}} \right)$$

## Thermodynamic Limits of Solar Cells

Understanding the fundamental limits in the energy conversion process of solar cells and determining a potential upper limit of the energy conversion efficiency is essential in developing high-efficiency solar cells [41–43]. The limit of the energy conversion efficiency of a solar cell can be derived by using the first and second laws of thermodynamics. In the first model, the solar conversion process is described as a heat engine, which converts the energy emitted by the sun in useable work. The energy is absorbed by a solar cell, which is described as an absorber at ambient temperature. The energy emitted by the sun is transferred to the solar cell and converted by the solar cell without creating entropy. Hence the conversion process is described by a reversible Carnot heat engine [44, 45]. The Carnot model is described in Sect. 3.1. Landsberg expanded the model by taking reflection losses and entropy generation into account. Details are provided in Sect. 3.2. Shockley and Queisser were the first to apply thermodynamics to a solar cell described as semiconductor device [5]. They introduced the concept of an ultimate solar cell energy conversion efficiency. In this model, the solar cell is described by a semiconductor with a bandgap. Hence large fractions of the incident light are lost due to thermalization and optical losses. Furthermore, it is assumed that the energy conversion process is free of recombination losses. Details are described in Sect. 3.3. In the next step, Shockley and Queisser expanded their model and took radiative recombination into account. The derived limit is commonly called the detailed balance or Shockley and Queisser limit. The model is described in Sect. 3.4. The solar cell is described as an ideal solar cell. Only the bandgap of the semiconductor is considered as a parameter in the description of the solar cell. Several authors expanded the model of Shockley and Queisser to describe special types of solar cells or consider charge transport and optical properties of materials [6, 46, 47]. The influence of charge transport processes on the detailed balance limit is described in Sect. 3.5, while the link between the detailed balance limit and the optics of a solar cell are described in Sect. 3.6. Besides the thermodynamics, the Yablonovitch limit sets an additional detailed balance limit, which must be considered. A brief introduction is provided in Sect. 3.6. Finally, the detailed balance model proposed by Shockley and Queisser is applied to tandem solar cells in Sect. 3.7.

### The Carnot Conversion Efficiency Limit

**Fig. 2:**
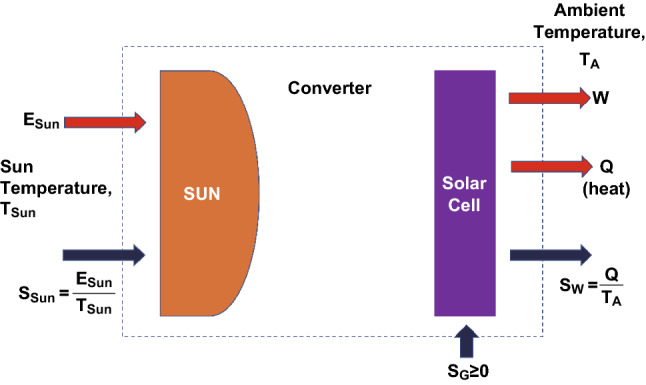
Schematic sketch of a solar cell represented by a Carnot reversible heat engine

The most fundamental energy conversion efficiency limit for a solar cell is the Carnot limit, which describes a solar cell as a heat engine as shown in Fig. 2. The input parameters are *E*_Sun_ and *S*_Sun_, where *E*_Sun_ and *S*_Sun_ are the heat flux and entropy flux coming from the sun. *T*_sun_ is the temperature of the sun which is assumed to be 6000 K. The entropy flux is given by *E*_sun_/*T*_sun_. The output parameters of the solar cell are represented by an energy flux in the form of useable work W and the heat flux Q emitted to the ambient. S_W_ is the entropy due to the generated heat energy and *T*_A_ is the ambient temperature. According to the first law of thermodynamics, the system can be described by Eq. 5:

5 $$E_{\text{Sun}} = W + Q,$$

where *E*_Sun_ is the input radiation energy from the sun, W and Q are the output work and heat energy, respectively. Accordingly, the equation of the heat flux can be expressed as Eq. 6:

6 $$S_{\text{Sun}} + S_{\text{G}} = S_{\text{W}}$$

where *S*_Sun_ is the entropy from the sun, while *S*_G_ is the entropy caused by transmission, absorption, and conversion of the sunlight. *S*_W_ is the entropy due to heat loss. It is assumed that no entropy is generated during the transmission, absorption or conversion of the sunlight. Hence *S*_G_ is assumed to be zero and the process is a reversible energy conversion process. The energy conversion efficiency can be determined by Eq. 7:

7 $$\eta_{C} = \frac{W}{{E_{\text{Sun}} }} = \frac{{E_{\text{Sun}} - Q}}{{E_{\text{Sun}} }} = 1 - \frac{Q}{{E_{\text{Sun}} }}$$

Because *S*_G_ is equal to zero, Eq. 5 can be rewritten by *E*_sun_/*T*_sun_ = *Q*/*T*_A_, so that the energy conversion efficiency can be expressed only by the input and output temperature.

8 $$\eta_{\text{C}} = 1 - \frac{{T_{\text{A}} }}{{T_{\text{Sun}} }}$$

Equation 8 defines the upper limit of the energy conversion process using the Carnot model.

The final expression of the energy conversion efficiency does not require or provide any information about the potential realization of such a converter. Furthermore, the calculations assumed that no entropy generation occurs during the transmission, absorption or conversion of the sunlight. However, Planck showed already at the beginning of the twentieth century, that an energy transfer between two blackbodies involves unavoidable entropy generation [3, 41, 48]. Landsberg tried to account for these entropy losses.

### Landsberg Conversion Efficiency Limit

**Fig. 3:**
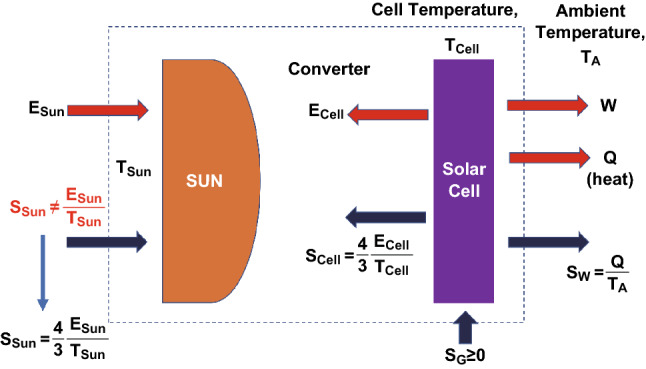
Schematic diagram of the Landsberg model for a solar converter

Landsberg calculated an energy conversion efficiency limit assuming that the sun and the solar cell are described as blackbodies with entropy losses, which means that the transmission, generation, and conversion lead to an entropy loss [41]. Furthermore, the input and output heat fluxes are replaced by input and output radiation energies. The schematic sketch of the Landsberg solar converter is illustrated in Fig. 3. The emission of an ideal blackbody is described by the Stefan Boltzmann law. According to the Stefan Boltzmann law, the radiation energy is given by Eq. 9:

9 $$E = \sigma_{\text{SB}} T^{4}$$

where *T* is the temperature of the blackbody and σ_SB_ is the Boltzmann constant. The entropy of the system can be calculated by solving the fundamental equation of thermodynamics TdS = dE, which leads to *S* = 4 × *E*/3/*T*, so that the entropy of the sun and the solar cell is given by Eq. 10:

10 $$S_{\text{Sun}} = \frac{4}{3}\frac{{E_{\text{Sun}} }}{{T_{\text{Sun}} }}$$

and Eq. 11:

11 $$S_{\text{Cell}} = \frac{4}{3}\frac{{E_{\text{Cell}} }}{{T_{\text{Cell}} }}$$

where *E*_Cell_ and S_Cell_ are the energy and entropy due to radiation, respectively. Now, Eqs. 5 and 6 become:

12 $$E_{\text{Sun}} = W + Q + E_{\text{Cell}}$$

13 $$S_{\text{Sun}} = S_{\text{W}} + S_{\text{Cell}} - S_{\text{G}}$$

The following expression (Eq. 14) can be derived, if we assume that the solar cell temperature (*T*_Cell_) is equal to the ambient temperature (*T*_A_):

14 $$\eta_{\text{L}} = \frac{W}{{E_{\text{Sun}} }} = \frac{{E_{\text{Sun}} - Q - E_{\text{Cell}} }}{{E_{\text{Sun}} }} = 1 - \frac{Q}{{E_{\text{Sun}} }} - \frac{{E_{\text{Cell}} }}{{E_{\text{Sun}} }} = 1 - \frac{Q}{{E_{\text{Sun}} }} - \frac{{T_{\text{cell}}^{4} }}{{T_{\text{Sun}}^{4} }}$$

The general solution of the Landsberg conversion efficiency is given by Eq. 15 [49, 50]:

15 $$\eta_{\text{L}} = 1 - \frac{{T_{\text{cell}}^{4} }}{{T_{\text{Sun}}^{4} }} - \frac{4}{3}\frac{{T_{\text{A}} }}{{T_{\text{Sun}} }} - \frac{4}{3}\frac{{T_{\text{A}} }}{{T_{\text{Cell}} }} \times \left( {\frac{{T_{\text{cell}}^{4} }}{{T_{\text{Sun}}^{4} }}} \right)$$

**Fig. 4:**
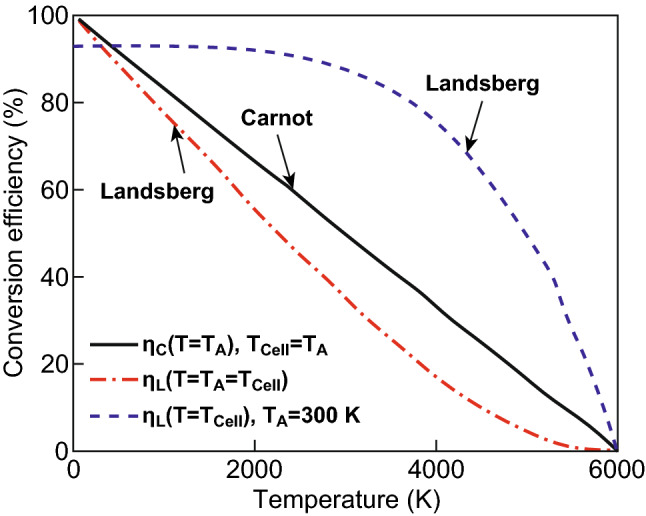
Temperature dependent Carnot and Landsberg efficiencies for solar energy conversion

The Landsberg conversion efficiency is plotted in Fig. 4 together with the Carnot limit. The Landsberg limit is plotted for two cases. In the first case, it is assumed that the temperature of the solar cell and the environment are equal and $T_{\text{Cell}} \approx T_{\text{A}}$. This is realistic for low solar cell temperatures, while the assumption is not realistic for high solar cell temperatures. In a second case, it is assumed that the ambient temperature is constant at *T*_A_ = 300 K, while the conversion temperature is varied. The conversion efficiency as a function of the temperature is illustrated in Fig. 4. According to the Carnot model, the upper conversion efficiency is limited to 95% assuming an ambient temperature of 300 K. The Landsberg model provides an upper limit of 93%. The energy conversion efficiency is zero if the converter and ambient temperature are equal to the sun temperature [50–52].

### The Ultimate Solar Cell Conversion Efficiency Limit

So far, the solar cell has been described by a general blackbody. Now we will assume that the solar cell is described by a single-junction solar cell, which consists of a semiconductor with a constant bandgap. The ultimate conversion efficiency represents a theoretical energy conversion efficiency limit of a semiconductor-based solar cell. Photons with photon energies larger than or equal to the bandgap are absorbed. Photons with energies smaller than the bandgap are not absorbed. It is assumed that photogenerated electron/hole pairs are collected. Recombination of electron/hole pairs is not considered. Only thermalization and absorption losses are considered. Absorption losses occur for photon energies smaller than the bandgap and thermalization losses occur for energies larger than the bandgap [41]. The photon flux of the sun, which is absorbed by the solar cell, is given by Eq. 16 [5, 41]:

16 $$F_{\text{Cell}} \left( {T = T_{\text{Sun}} } \right) = \frac{2\pi }{{h^{3} c^{2} }}\int_{{E_{\text{g}} }}^{\infty } {\frac{{E^{2} {\text{d}}E}}{{\exp \left( {\frac{E}{{kT_{\text{Sun}} }}} \right) - 1}}}$$

where *h*, *c*, *k*, and *E*_G_ are Planck’s constant, speed of light, Boltzmann constant, and energy bandgap of the photovoltaic material. The photon flux can be approximated by Eq. 17:

17 $$F_{\text{Cell}} \left( {T = T_{\text{Sun}} } \right) \cong \frac{2\pi }{{h^{3} c^{2} }}\int_{{E_{\text{g}} }}^{\infty } {\exp \left( { - \frac{E}{{kT_{\text{Sun}} }}} \right)E^{2} {\text{d}}E = \int_{{E_{\text{g}} }}^{\infty } {\phi_{\text{Sun}} {\text{d}}E} }$$

where *ϕ*_sun_ is the blackbody radiation flux of the sun, which is given by Eq. 18:

18 $$\phi_{\text{Sun}} = \frac{2\pi }{{h^{3} c^{2} }} \times E^{2} \times \exp \left( { - \frac{E}{{kT_{\text{Sun}} }}} \right)$$

The photocurrent density of the solar cell is given by *J* = *q * ×  *F*_Cell_ (*T* = *T*_sun_). The electrical output power density of the solar cell is calculated by Eq. 19:

19 $$P_{\text{Out}} = J \times V = q \times F_{\text{Cell}} \left( {T = T_{\text{Sun}} } \right) \times \frac{{E_{\text{G}} }}{q} = F_{\text{Cell}} \left( {T = T_{\text{Sun}} } \right) \times E_{\text{g}}$$

The input sun power density is given by Eq. 20 [41]:

20 $$P_{\text{in}} = \frac{2\pi }{{h^{3} c^{2} }}\int_{0}^{\infty } {\frac{{E^{3} {\text{d}}E}}{{\exp \left( {\frac{E}{{kT_{\text{Sun}} }}} \right) - 1}} \cong \frac{{2\pi^{5} \left( {kT_{\text{Sun}} } \right)^{4} }}{{15h^{3} c^{2} }}}$$

**Fig. 5:**
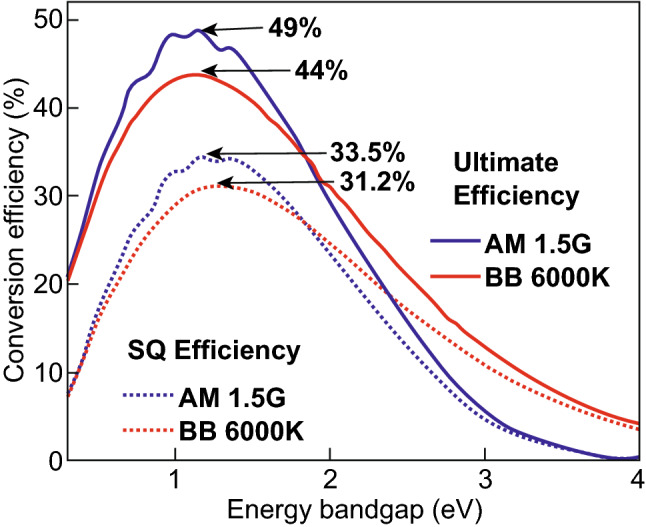
Ultimate conversion efficiency and Shockley–Queisser limit of single-junction solar cells as a function of the bandgap. A blackbody spectrum at 6000 K and an AM 1.5G spectrum were used for the calculations

Finally, the energy conversion efficiency of a solar cell is calculated by *η* = *P*_out_/*P*_in_. By using the blackbody spectrum (*T* = 6000 K) and AM 1.5 global spectrum, the solar cell exhibits a maximum of the ultimate conversion efficiency of 44% and 49%, respectively, for an optimal bandgap of 1.1 eV as shown in Fig. 5. These energy conversion efficiencies are significantly lower than the Carnot and Landsberg limits because of the two losses, absorption losses and thermalization losses, which are large for single-junction solar cells [41].

### Detailed Balance Limit or Shockley-Queisser Limit

The ultimate energy conversion efficiency limit derived by Shockley and Queisser does not consider radiative emission by the solar cell. Hence the ultimate conversion efficiency limit violates the Kirchhoff law. Shockley and Queisser expanded their model commonly called detailed balance limit or Shockley Queisser limit, by taking radiative recombination into account. Thermal generation and non-radiative recombination are not considered. The photon flux emitted by the solar cell can be described by blackbody radiation. The emission energy is given by *E* − *qV*, where *V* is the voltage applied to the solar cell [5, 41].

21 $$F_{\text{R}} \left( V \right) = \frac{2\pi }{{h^{3} c^{2} }}\int_{{E_{\text{G}} }}^{\infty } {\frac{{E^{2} }}{{\exp \left( {\frac{E - qV}{{kT_{\text{cell}} }}} \right) - 1}}{\text{d}}E}$$

The applied voltage is equal to the splitting of the Fermi levels *qV* = *E*_F_^n ^− *E*_F_^p^, where *E*_F_^n^ and *E*_F_^p^ are the majority of quasi-Fermi levels in the p- and n-region of the p–n junction. The quasi-Fermi levels are determined by the free carrier concentration, which is again determined by doping concentration, generation, and recombination of charges. With increasing photogeneration the quasi-Fermi levels shift closer to the conduction and valence bands and the open-circuit voltage is increased, while for recombination the quasi-Fermi levels shift away from the conduction and valence bands and the open-circuit voltage is reduced. The photon flux emitted by the solar cell can be approximated by Eq. 22:

22 $$F_{\text{R}} \left( V \right) \cong \frac{2\pi }{{h^{3} c^{2} }}\int_{{E_{\text{g}} }}^{\infty } {\exp \left( { - \frac{E - qV}{{kT_{\text{Cell}} }}} \right)E^{2} {\text{d}}E = \exp \left( {\frac{qV}{{kT_{\text{Cell}} }}} \right)\int_{{E_{\text{G}} }}^{\infty } {\phi_{\text{Cell}} {\text{d}}E} }$$

The equation can be simplified and the blackbody radiation flux *ϕ*_cell_ of the solar cell can be described by Eq. 23:

23 $$\phi_{\text{Cell}} = \frac{2\pi }{{h^{3} c^{2} }}E^{2} \exp \left( { - \frac{E}{{kT_{\text{Cell}} }}} \right)$$

so that the photon flux at zero applied voltage is given by Eq. 24:

24 $$F_{{{\text{R}}0}} = \int_{{E_{\text{G}} }}^{\infty } {\phi_{\text{Cell}} {\text{d}}E}$$

Combining Eqs. 22–24 allows for describing the photon flux as a function of the applied voltage.

25 $$F_{\text{R}} \left( V \right) = F_{{{\text{R}}0}} \times \exp \left( {\frac{qV}{{kT_{\text{Cell}} }}} \right)$$

The expression for the total current density is given by Eq. 26 [5, 46]:

26 $$J\left( V \right) = q \times \left[ {F_{\text{cell}} - F_{\text{R}} \left( V \right)} \right]$$

The short-circuit current and open-circuit voltage can be calculated by Eq. 27:

27 $$J_{\text{SC}} = q \times \left[ {F_{\text{cell}} - F_{{{\text{R}}0}} } \right]$$

and Eq. 28:

28 $$V_{\text{OC}} = \frac{{kT_{\text{Cell}} }}{q} \times \ln \left( {\frac{{F_{\text{cell}} }}{{F_{{{\text{R}}0}} }}} \right)$$

The fill factor and energy conversion efficiency of the solar cell are given by Eq. 29:

29 $$FF = \frac{{\hbox{max} \left[ {J\left( V \right) \times V} \right]}}{{J_{\text{SC}} \times V_{\text{OC}} }}$$

and Eq. 30:

30 $$\eta = \frac{{\hbox{max} \left[ {J\left( V \right) \times V} \right]}}{{P_{\text{in}} }}$$

**Fig. 6:**
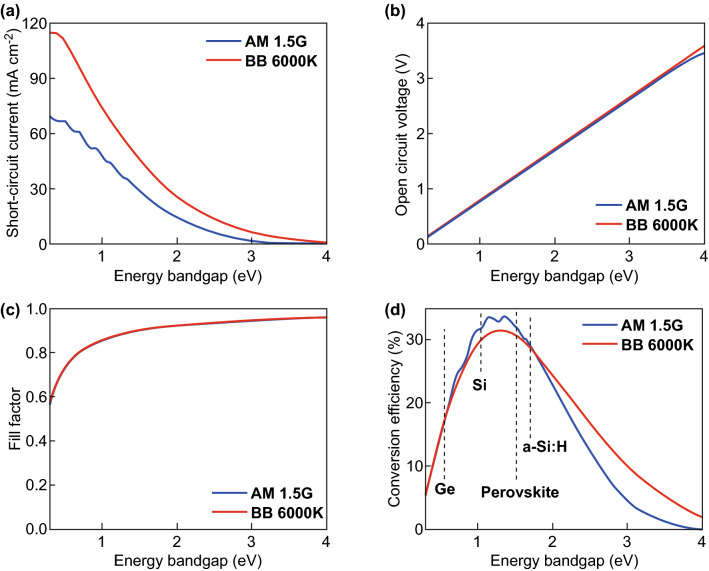
Detailed Balance limit (Shockley–Queisser limit) for **a** short-circuit current, **b** open-circuit voltage, **c** fill factor, and **d** conversion efficiency under AM 1.5G spectrum with blackbody spectrum at 6000 K

The short-circuit current density, open-circuit voltage, fill factor and energy conversion efficiency as a function of the bandgap are shown in Fig. 6 for blackbody radiation and an AM 1.5G sun spectrum. The short-circuit current density increases, while the open-circuit voltage decreases as a function of the bandgap. The optimal bandgap represents a trade-off between the short-circuit current density and the open-circuit voltage. The energy conversion as a function of the bandgap is provided in Fig. 6d. The energy conversion efficiency reaches a maximum value of ~ 33.5%. The maximum conversion efficiency is observed for a bandgap of 1.2–1.4 eV. A comparison of the ultimate energy conversion efficiency and the Shockley-Queisser conversion efficiency limit is plotted in Fig. 6. The additional loss of energy conversion efficiency is caused by radiative recombination. The ultimate conversion efficiency exhibits its maximum for 1.1 eV, while the Shockley-Queisser limit exhibits a maximum at 1.2–1.4 eV. The difference is approximately equal to *E*_G_-q × *V*_OC_, where *V*_OC_ is the open-circuit voltage according to the Shockley-Queisser limit (Eq. 28). In other words, considering radiative recombination losses leads to a shift of the optimal bandgap to larger bandgaps. Hence materials with appropriate bandgaps can be selected. The electronic and optical properties of the materials will determine if the material allows for reaching energy conversion efficiencies close to the Detailed balance limit [5].

### Detailed Balance and Charge Transport

The detailed balance limit commonly called Shockley Queisser limit assumes only radiative recombination. However, to provide a more realistic description additional generation/recombination processes must be considered. To derive the *J*(V) characteristic of a solar cell the following five processes (already stated in the original work of Shockley and Queisser) must be considered [5]:

1. Generation of electron–hole pairs by the illumination, *G*_Cell_.
2. Radiative recombination, *R*_R_.
3. Non-radiative generation processes or thermal generation, *G*_0_.
4. Non-radiative recombination, *R*_NR_.
5. Extraction of electron–hole pairs as current flow, *J*.

The steady-state *J*(V) characteristic of the solar cell taking the five process into account can be described by Eq. 31:

31 $$q \times d \times \left[ {G_{\text{Cell}} + G_{0} - R_{\text{R}} \left( V \right) - R_{\text{NR}} \left( V \right)} \right] - J = 0$$

where *d* is the thickness of the solar cell. The radiative and non-radiative recombination can be described by Eq. 32:

32a $$R_{\text{R}} \left( V \right) = R_{{{\text{R}}0}} \times \exp \left( {\frac{qV}{{kT_{\text{Cell}} }}} \right)$$

32b $$R_{\text{NR}} \left( V \right) = R_{{{\text{NR}}0}} \times \exp \left( {\frac{qV}{{kT_{\text{Cell}} }}} \right)$$

where *R*_R0_ and *R*_NR0_ are the radiative and non-radiative recombination rates for an applied voltage of zero volts. At full sunlight, the generation *G*_Cell_ is distinctly larger than the non-radiative generation rate or thermal generation, *G*_0_, so that we will not consider a non-radiative generation rate or thermal generation in future calculations. If we further assume that *R*_NR_ is negligible, we get the following expressing (Eq. 33) for the open-circuit voltage

33 $$V_{\text{OC}} = \frac{{kT_{\text{Cell}} }}{q} \times \ln \left( {\frac{{G_{\text{Cell}} }}{{R_{{{\text{R}}0}} }}} \right)$$

The expression follows the classical description of the Shockley-Queisser limit, which is described in Sect. 3.4. If we consider non-radiative recombination, we receive Eq. 34 for the open-circuit voltage

34 $$V_{\text{OC}} = \frac{{kT_{\text{Cell}} }}{q} \times \ln \left( {\frac{{G_{\text{phot}} }}{{R_{{{\text{R}}0}} + R_{\text{NR}} }}} \right)$$

Instead of expressing the open-circuit voltage in terms of the generation rate we will describe the open-circuit voltage in terms of the short-circuit current density and the saturation current density, which leads to Eq. 35,

35 $$V_{\text{OC}} = \frac{{kT_{\text{Cell}} }}{q} \times \ln \left( {\frac{{J_{\text{SC}} }}{{J_{0} }}} \right) = \frac{{kT_{\text{Cell}} }}{q} \times \ln \left( {\frac{{J_{\text{SC}} }}{{J_{ 0}^{\text{rad}} + J_{ 0}^{\text{non-rad}} }}} \right)$$

where the saturation current density, *J*_0_, is the sum of the radiative recombination saturation current density, *J*_0_^rad^, and the non-radiative recombination saturation current density, *J*_0_^non-rad^. The equation can be rewritten by using the logarithm laws, so that the first term, *V*_0_^rad^, considers only radiative recombination.

36 $$V_{\text{OC}} = \frac{{kT_{\text{C}} }}{q} \times \left[ {\ln \left( {\frac{{J_{\text{SC}} }}{{J_{ 0}^{\text{rad}} }}} \right) + \ln \left( {\frac{{J_{ 0}^{\text{rad}} }}{{J_{ 0}^{\text{rad}} + J_{ 0}^{\text{non-rad}} }}} \right)} \right] = V_{\text{OC}}^{\text{rad}} + \frac{{kT_{\text{C}} }}{q}\ln \left( {\frac{{J_{ 0}^{\text{rad}} }}{{J_{ 0}^{\text{rad}} + J_{ 0}^{\text{non-rad}} }}} \right)$$

The first term is equal to the open-circuit voltage derived in Eqs. 28 and 35. In both cases, only radiative recombination is considered when calculating the open-circuit voltage. The second term contains all entropic losses that are related to non-radiative recombination and parasitic absorption of photons in the solar cell. The ratio of the radiative recombination saturation current density and the total saturation current density can be expressed as the quantum efficiency of a p–n junction operating as a light emitting diode (LED). QE_LED_ is the external quantum efficiency of the p–n junction operating as LED.

37 $${\text{QE}}_{\text{LED}} = \frac{{J_{ 0}^{\text{rad}} }}{{J_{0} }} = \frac{{J_{ 0}^{\text{rad}} }}{{J_{ 0}^{\text{rad}} + J_{ 0}^{\text{non-rad}} }}$$

Here, we distinguish the saturation current (*J*_0_^rad^) that leads to the emission of photons, and the saturation current (*J*_0_^non-rad^) that does not lead to photon emission. The final expression of the open-circuit voltage is given by Eq. 38:

38 $$V_{\text{OC}} = V_{\text{OC}}^{\text{rad}} + \frac{{kT_{\text{C}} }}{q} \times \ln \left( {{\text{QE}}_{\text{LED}} } \right)$$

If the non-radiative saturation current density is zero, the external quantum efficiency of the LED is equal to one and Eq. 38 is equal to Eq. 28, which was already calculated in the original work of Shockley and Queisser (Sect. 3.4). Due to detailed balance, the emission and absorption properties of a solar cell are related. However, the relationship between absorption and emission in a semiconductor is only valid if the quasi-Fermi level splitting is constant over the whole volume of the absorber [2, 53]. The relationship between the quantum efficiency and absorption of the solar cell with the short-circuit current radiative and non-radiative saturation current is given in Eqs. 39 and 40.

39 $$J_{\text{SC}} = q\varepsilon_{\text{in}} \int_{0}^{\infty } {QE_{\text{cell}} \left( E \right) \times \phi_{\text{Sun}} \left( E \right){\text{d}}E}$$

40 $$J_{0}^{\text{rad}} = q\varepsilon_{\text{out}} \int_{0}^{\infty } {{\text{QE}}_{\text{cell}} \left( E \right) \times \phi_{\text{cell}} \left( E \right){\text{d}}E}$$

where *ε*_in_ and *ε*_out_ are the etendue describing the incoupling and outcoupling of light. By considering that *A*(*E*) + *R*(*E*) = 1 and *A*(*E*) = QE_cell_(*E*) + *A*_para_(*E*), where *A*(*E*) is the total absorption of the solar cell, *R*(*E*) is the total reflection for the solar cell and *A*_para_(*E*) is the parasitic losses, an expression for the non-radiative recombination can be derived by Eq. 41:

41 $$J_{0}^{\text{non-rad}} = q\varepsilon_{\text{out}} \int_{0}^{\infty } {\left[ {A_{\text{Cell}} \left( E \right) - {\text{QE}}_{\text{cell}} \left( E \right) - R\left( E \right)} \right] \times \phi_{\text{cell}} \left( E \right){\text{d}}E}$$

Equations 39–41 provide some guideline to maximize the energy conversion efficiency of a real solar cell. By increasing the quantum efficiency of a solar cell, the short-circuit current density and the radiative saturation current density is increased. Furthermore, the QE_LED_ is increased. Hence the open-circuit voltage is increased too. Ideally, the QE_LED_ is approaching unity, so that the open circuit is converging toward the maximal value as stated in Eqs. 28 and 35. This can be achieved by minimizing reflection of the solar cell due to improved light incoupling or light trapping or minimizing parasitic losses. Parasitic optical losses or non-radiative losses lead to a drop of the QE_Cell_ and drop of QE_LED_. Both effects lead to a lowering of the open-circuit voltage. Hence such losses should be minimized or avoided. The optics of a solar cell influences all three parameters, short-circuit current, open-circuit voltage and fill factor. By optimizing the optics all three parameters can be increased. On the other hand, non-radiative losses have not only a negative effect on the short-circuit current. The open-circuit voltage and the fill factor are negatively affected too. In the following, the interplay between detailed balance and optics will be described.

### Detailed Balance and Photon Management

The aim of photon management in a solar cell is to increase the QE_cell_ by minimizing reflection and parasitic optical losses. One way to increase QE_cell_ is light trapping. In this case, the optical path length of light in the solar cell is increased by the design of the solar cell or a specific light trapping structure, which is integrated into the solar cell. However, the maximal optical path length enhancement is limited to 2*n*^2^, where n is the refractive index of the absorber material of the solar cell. Hence the quantum efficiency of a semiconducting slab is limited to (as Eq. 42)

42 $${\text{QE}}_{\text{cell}} \left( E \right) \cong A_{\text{Cell}} \left( E \right) = 1 - \exp \left( { - 4 \times \alpha \left( E \right) \times n^{2} \left( E \right) \times d} \right)$$

where *α*(*E*) and *n*(*E*) are the absorption coefficient and the refractive index. For short wavelengths, the penetration depth of the photons is typically smaller than twice the thickness of the solar cell. For long wavelengths, the penetration depth might exceed twice the thickness of the solar cell. Hence light trapping is of importance for long wavelengths. Equation 42 can be developed in a Taylor series. For long wavelength and weak absorbing materials is 1/($4 \times n\left( E \right) \times d$) ≫ *α*(E), so that the absorbance for can be approximated by Eq. 43:

43 $$QE_{\text{cell}} \left( E \right) \cong 4 \times \alpha \left( E \right) \times n^{2} \left( E \right) \times d$$

The absorption limit derived by Yablonovitch et al. represents a ray optics or geometrical optics limit. The limit is valid for absorber thicknesses much larger than the wavelength of the incident light. The same applies to the dimensions of the light trapping textures. The size of the surface features must be distinctly larger than the incident wavelengths. For thin-film solar cells, these assumptions might not be fulfilled. Yu et al. have shown that higher quantum efficiencies and short-circuit currents can be achieved by using wave optics [54, 55]. Furthermore, the authors proposed potential solar cells with higher short-circuit currents [54–56]. In general, a variety of optical concepts can be applied to increase the quantum efficiency of solar cells and/or reduce the material consumption in the solar cell fabrication process. The concepts can be divided into three optical domains distinguished by the size of the surface features or surface textures. Features distinctly smaller than the optical wavelength can be used to minimize the reflection at an interface. The optics can be described by effective medium theory. Most of the used structures act as broadband anti-reflection coatings to improve the incoupling of light in the solar cell. If the feature size is comparable to the wavelengths of the incident light, diffraction might be used to increase the optical path lengths of light in the solar cell. This concept is often applied to silicon thin-film solar cells. Silicon, being an indirect semiconductor with a low absorption coefficient close to the bandgap requires the use of light trapping to reach short-circuit currents close to the theoretical limits. In the case of crystalline silicon wafer-based solar cells, refraction of the incident light is usually used to enhance the optical path length in the solar cell. The textures are usually formed by anisotropic etching of silicon wafers. Table 1 describes the optical wave propagation and photon management mechanism in solar cells.

**Table 1:** Optical wave propagation and photon management mechanisms in solar cells

Feature size	Period ≪ wavelength	Period ≅ wavelength	Period ≫ wavelength
Physical effect	Formation of a refractive index gradient	Diffraction of light	Refraction of light
Description of optical wave propagation	Effective medium theory	Diffraction theory or numerical simulation	Ray or geometric optics
Potential application	Broadband anti-reflection coating	Light trapping in thin-film solar cell	Light trapping in bulk solar cell

**Fig. 7:**
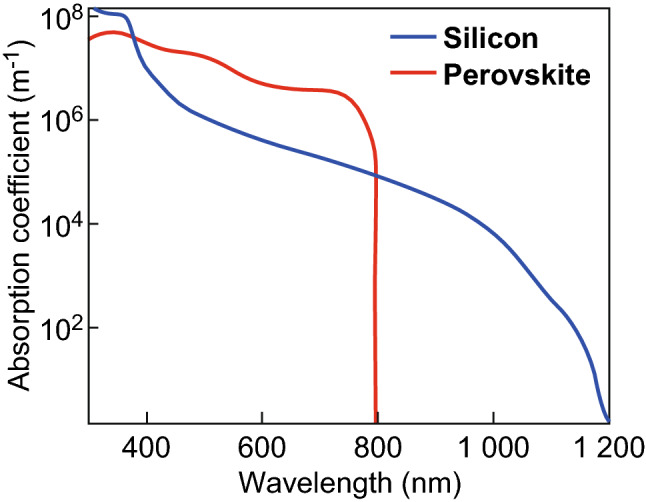
Absorption coefficients of crystalline silicon and MAPbI_3_ perovskite

In this study, we have investigated the optics of perovskite single-junction solar cells and perovskite/silicon tandem solar cells. The absorption coefficients of perovskite and crystalline silicon are shown in Fig. 7. The perovskite material system is a direct semiconductor. The material exhibits a high absorption coefficient and a low penetration depth. Furthermore, the diffusion length is larger than the penetration depths. Hence, light trapping is not required to increase the quantum efficiency and short-circuit current of the solar cell. The emphasis must be on minimizing reflection losses and parasitic losses in the solar cell. Crystalline silicon is an indirect semiconductor, and the penetration depth close to the bandgap is larger than the thickness of a typical solar cell. Hence light trapping is applied to increase the quantum efficiency and short-circuit current. Typically, the surface of a crystalline solar cell is characterized by large surface features, which support the refraction of the incident light. Photon management in silicon solar cells is more complex than photon management in perovskite solar cells. In the case of a silicon solar cell, the incident light must be efficiently coupled in the solar cell, the light in the solar cell must be confined by a light trapping structure, and lastly, the light trapping structure must be design in a way that parasitic optical losses are kept small. In the case of a perovskite solar cell, the incident light must be efficiently coupled in the solar cell and optical losses must be minimized. In Sect. 4, the described guidelines will be used to design solar cells with high short-circuit currents and energy conversion efficiencies. Finite-difference time-domain (FDTD) simulations will be used to simulate the optical wave propagation.

### Detailed Balance Limit of Tandem Solar Cells

**Fig. 8:**
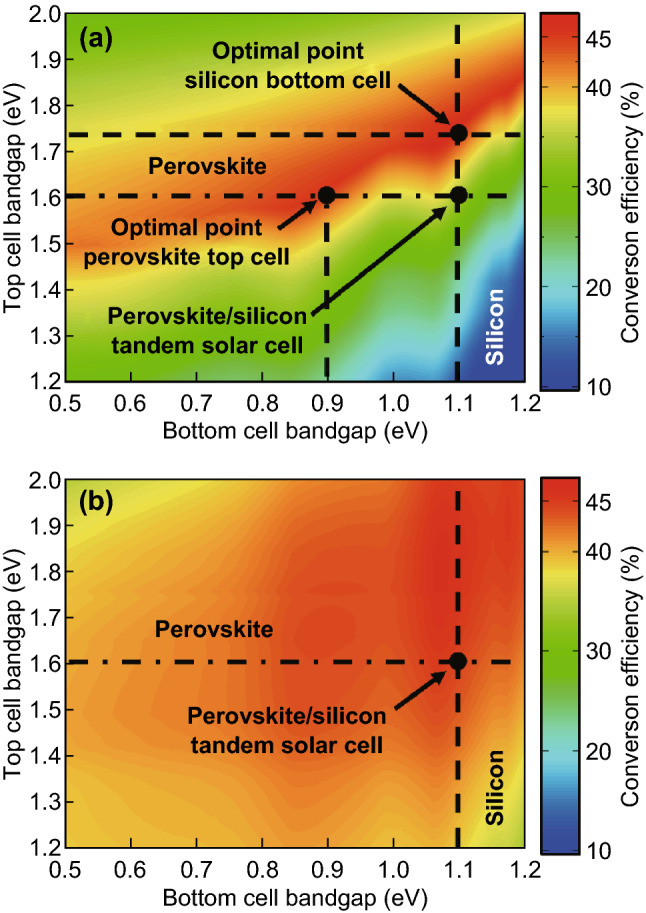
Detailed balance conversion efficiency limit for **a** 2-terminal and **b** 4-terminal tandem solar cells

Detailed balance limit calculations are not only restricted to a single-junction solar cell. The calculations can be applied to tandem or multi-junction solar cell. The first detailed balance calculation for tandem solar cells was published by De Vos [6]. A general description of the detailed balance theory for multi-junction solar cells is provided by Green [16]. Here we will discuss the detailed balance limit of tandem solar cells and its implications for perovskite/silicon tandem solar cells. In general, a tandem solar cell can be operated as two and four-terminal devices. Plots of the energy conversion efficiency of two and four-terminal tandem solar cells are provided in Fig. 8a, b. In the case of a four-terminal device, the incident light is divided into two diodes, while both diodes are electrically independent. For a variety of combinations of bandgaps, an energy conversion efficiency exceeding 40% can be reached. The electrical output power generated by both diodes is calculated independently and added when calculating the energy conversion efficiency. Only the incident light must be divided among the two solar cells. The energy conversion efficiency of a two-terminal device or a serial connected tandem solar cell is shown in Fig. 8a. The energy conversion efficiency is mainly determined by the short-circuit current of the tandem solar cell. The total short-circuit current is equal to the short-circuit current of the bottom solar cell if the short-circuit current of the bottom diode is smaller than the short-circuit current of the top diode. The total short-circuit current is determined by the short-circuit current of the top diode if the short-circuit current is larger than the short-circuit current of the bottom diode. The short-circuit current of a tandem solar is matched if the short-circuit current of the top and the bottom diode is equal or almost equal. The energy conversion efficiency of a tandem solar cell is maximized for a combination of top and bottom solar cells with matched bandgaps. If the right combination of bandgaps of the top and bottom solar cell is selected, and the short-circuit current of the two-terminal tandem solar cell is matched and the two-terminal tandem solar cells can reach energy conversion efficiencies equal to the four-terminal tandem solar cells. A maximal energy conversion efficiency can be reached if the bandgap of the top cell is equal to *E*_G_top_ = 0.5 × *E*_G_bot_ + 1.15 eV. Crystalline silicon solar cells dominate commercial solar cell technology. The energy conversion efficiency of a tandem solar cell with a crystalline silicon bottom solar cell is maximized if the bandgap of the top diode is ~ 1.725 eV. The combination allows for a maximal energy conversion efficiency of ~ 43%. In the current study, we use perovskite (MAPbI_3_) as an absorber with a bandgap of ~ 1.6 eV. The maximal energy conversion efficiency of a perovskite/silicon tandem solar cell is ~ 33%. If we start with a perovskite top cell with a bandgap of 1.6 eV the optimal bandgap of the bottom solar cell is 0.9 eV, which allows for realizing an upper energy conversion efficiency of ~ 44%.

## Photon Management in Solar Cells

Perovskites have gained considerable attention as a photovoltaic material [25–27]. Since 2009 the energy conversion efficiency of single-junction perovskite solar cells has been increased to over 22% [33–37]. Furthermore, perovskites are a promising material system for the implementation of tandem or multi-junction solar cells. For example, perovskite/crystalline silicon tandem solar cells allow for reaching potentially high energy conversion efficiencies while potentially maintaining low fabrication cost. Detailed balance calculations presented in the previous section show that energy conversion efficiencies higher than the best single-junction solar cells could be reached by transitioning toward tandem solar cells. The combination of crystalline silicon and the perovskite material system is a very good match. So far, perovskite/crystalline silicon tandem solar cells in two-terminal configuration and four-terminal configurations have reached energy conversion efficiencies of 25.2% and 26.4%, respectively [8, 57]. Recently, the energy conversion efficiency of the perovskite/silicon tandem solar cell has reached to 28% [38], which is reported by Oxford PV; however, detailed on the used solar cell structure has not been revealed yet. The bandgap of the perovskite material system can be controlled over a wide range [58–61]. In this study, we will use CH_3_NH_3_PbI_3_, the best-studied material out of the group of perovskites. CH_3_NH_3_PbI_3_ exhibits a bandgap of ~ 1.6 eV. Up to now, most of the research on perovskite materials and solar cells has focused on understanding electronic charge transport properties [24, 62–67]. Much less effort has been devoted to the optimization of the optical properties of perovskite solar cells [68–70]. The high extinction coefficient and large diffusion length allow for realizing solar cells with high short-circuit current densities and energy conversion efficiencies [22, 71–74]. The optics of the solar cell can be improved by enhanced light incoupling and minimizing optical losses. The largest gains can be achieved by an improved light incoupling [75, 76]. A variety of structures have been investigated that exhibit improved light incoupling. Here we will focus on moth eye textures, which exhibit excellent in and out coupling properties.

### Device Design and Material Properties

The aim is to realize perovskite/silicon tandem solar cells with a high energy conversion efficiency. In the case of a perovskite/silicon tandem solar cell, the perovskite top solar cell must be fabricated on top of the crystalline silicon bottom solar cell. Therefore, the tandem solar cell is a solar cell in a substrate configuration. Hence, we will focus in this study only on solar cells in substrate configuration. In the first step, we will investigate perovskite single-junction solar cells before moving to a perovskite/silicon tandem solar cell.

The perovskite single-junction solar cell consists of a hydrogen doped tin oxide (IOH)/Nickel oxide (NiO) double layer, a perovskite (CH_3_NH_3_PbI_3_) layer, Zinc oxide (ZnO) interlayer, and an aluminum reflector. All charge transport and charge blocking layers used in this study are metal oxide layers. These layers can be deposited by physical (PVD) or chemical vapor deposition (CVD). We decided to avoid spin coated transport and charge blocking layers because the layers must be prepared on textured substrates [77]. PVD and CVD seem more suited for the deposition of uniform films on textured substrates. Nevertheless, a variety of charge transport and charge blocking layers has been suggested and successfully implemented including well-established materials like Spiro-MeOTAD and TiO_2_ or novel materials like graphene oxide or Cu-phthalocyanine [78–83]. Furthermore, it might be necessary to use multiple layers to fulfill all the requirements of the contact layers.

**Fig. 9:**
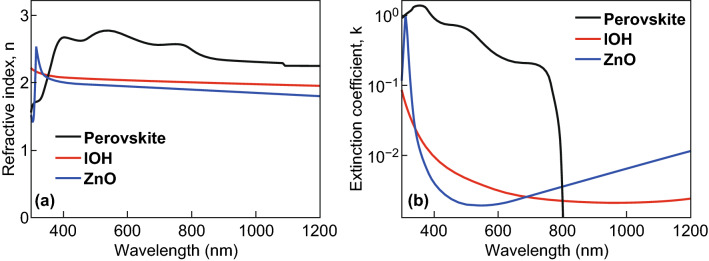
**a** Refractive Indices and **b** Extinction coefficients of Perovskite (CH_3_NH_3_PbI_3_), IOH, and ZnO materials

The NiO film is used as a hole transport and an electron blocking layer. However, NiO films exhibit a low hole charge carrier mobility, so that the lateral conductivity of the films is too low to realize solar cells with low sheet resistance and high fill factor. Highly doped NiO films exhibit high absorption losses, so that a double layer of IOH/NiO is used. IOH exhibits a high electron charge carrier mobility, and NiO exhibits a high work function which permits to achieve efficient hole injections and provides good lateral charge transport, so that solar cells with low series resistance and high fill factor can be achieved [84, 85]. The IOH/NiO double layer forms a tunnel junction. Similar combinations of materials like ITO/NiO were used in literature to experimentally realize perovskite single-junction solar cells with high energy conversion efficiency [86, 87]. The NiO is only 5 nm thick so that the absorption by the layer is low. Hence we have not considered the NiO layer in the optical simulations [77, 88]. The optical constants used for the simulation were taken from the literature [67, 74, 89]. Figure 9 illustrates the complex refractive indices and extinction coefficients of IOH, perovskite (CH_3_NH_3_PbI_3_), and ZnO. The perovskite material system and the metal oxide contact layers have a comparable refractive index. Hence the reflection at the perovskite/metal oxide interface is low and the entire layer stack exhibits a comparable refractive index [90].

### Optical Simulation Method

To investigate the optics of perovskite single-junction and perovskite/silicon tandem solar cells, three-dimensional (3D) finite-difference time-domain (FDTD) simulations have been performed [77, 91–93]. The complex refractive indices of the used materials are provided in Fig. 9 [71, 77]. The refractive index of the Al back reflector is taken from Ref. [94]. Circularly polarized light with an input amplitude of 1 V m^−1^ is used for the numerical simulations. For a single-junction perovskite solar cell, the electric field is calculated from 300 to 800 nm while for a perovskite/silicon tandem solar cell the spectral range from 300 to 1200 nm was used. Based on the electric field distribution the power density and the short-circuit current is calculated. Details on the calculation are provided in the literature [95–97]. It is assumed that only the electron/hole pairs absorbed by the absorber layers of the solar cell contribute to the quantum efficiency and the short-circuit current density. Light absorbed by all other layers including the contact layers is lost due to non-radiative recombination. Furthermore, it is assumed that the collection efficiency is 100% because the thickness of the absorber layer is smaller than diffusion lengths of the photogenerated charges. Hence, the calculated quantum efficiency represents an upper limit.

### Perovskite Single-Junction Solar Cells

#### Flat Interface

**Fig. 10:**
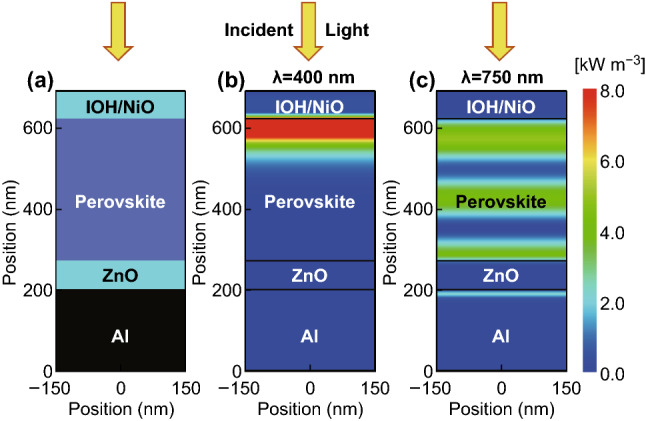
**a** Schematic cross section, and simulated power density under monochromatic illumination of wavelength **b** 400 nm and **c** 750 nm for the flat perovskite solar cell

**Fig. 11:**
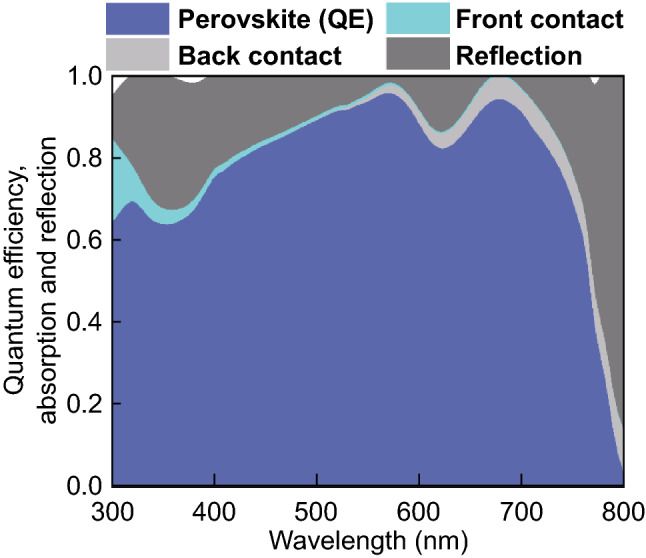
Quantum efficiency and absorption of individual layers of a planar perovskite solar cell

We start with the investigation of a flat or planar perovskite single-junction solar cell. Figure 10a shows the schematic cross section of a perovskite solar cell on a smooth or flat substrate. The solar cell consists of a 70 nm front hydrogen doped tin oxide (IOH)/Nickel oxide (NiO) double layer, a 350 nm perovskite (CH_3_NH_3_PbI_3_) material, a 70 nm Zinc oxide (ZnO) layer, and an aluminum layer as a reflector. The time average power density for the incident wavelength of 400 and 750 nm is provided in Fig. 10b, c. Due to the high absorption coefficient of the perovskite material for the short wavelengths (400 nm), most photons are absorbed within a couple of tens of nanometers of the perovskite film. For long wavelengths (750 nm) the absorption coefficient is reduced, and a certain fraction of the incident light reaches the back reflector, where the light is a reflection so that a standing wave is formed in front of the back reflector. The quantum efficiency of the perovskite solar cell is shown in Fig. 11. The quantum efficiency is plotted together with the absorption of the front and back contact and the reflection of the solar cell. The perovskite material system exhibits a bandgap of ~ 1.6 eV, which results in an upper short-circuit current density of 26.9 mA cm^−2^. The short-circuit current density of the simulated single-junction perovskite solar cell is 21.4 mA cm^−2^. A short-circuit current of 5.5 mA cm^−2^ is lost due to absorption and reflection losses. The reflection accounts for an optical loss of 4.4 mA cm^−2^, which correspond to 16% of total short-circuit current density, while the absorption losses of the front and back contact account for 4% of the total short-circuit current density. The short-circuit current density can be distinctly improved by an improving coupling of the incident light in the solar cell.

#### Solar Cell with Moth Eye Texture

**Fig. 12:**
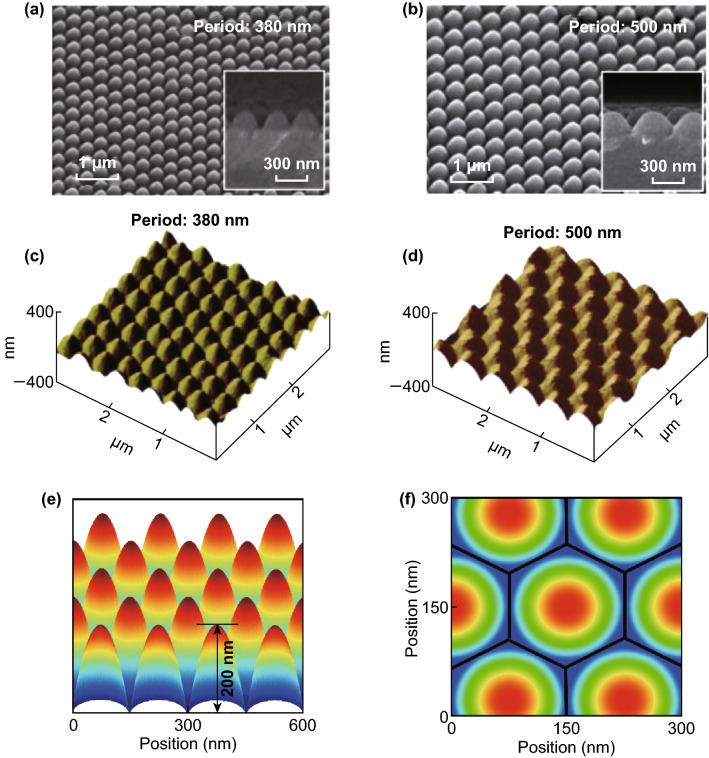
**a, b** SEM and **c, d** AFM images of a moth eye surface texture. Reproduced with permission from Ref. [101]. Copyright 2019, Elsevier. **e, f** Moth eye surface texture used for the optical simulation of the solar cells

**Fig. 13:**
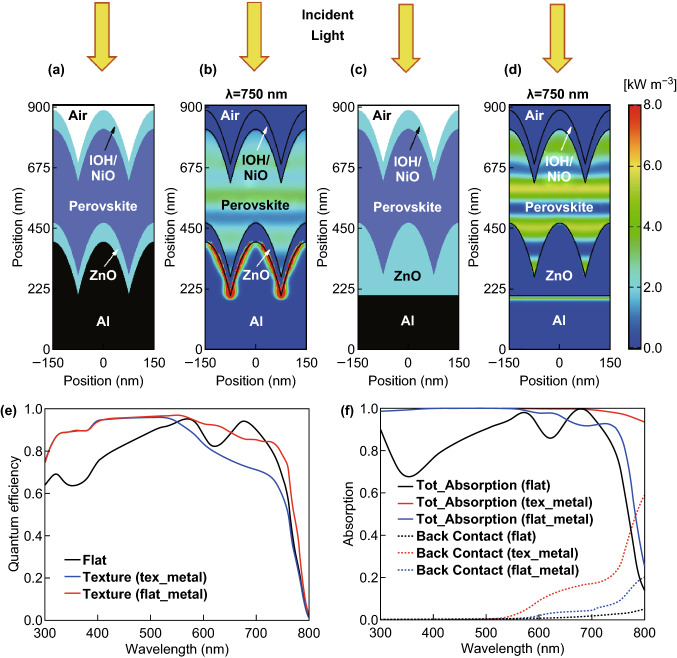
Schematic cross sections of a perovskite solar cell with integrated moth eye textures. **a** An aluminum contact with a moth eye texture is formed and all subsequent layers of the solar cell are formed on the textured metal contact. Hence, all layers of the solar cell exhibit a moth eye texture. **c** A moth eye textured interlayer is form on a flat or planar aluminum reflector. All layers are formed on the moth eye texturized interlayer. Hence, all layers except the aluminum back contact exhibits a moth eye texture, and **b, d** corresponding power density maps for an incident wavelength of 750 nm. Periods and heights of the moth eye structures are 150 and 200 nm, respectively. **e** Comparison of calculated quantum efficiency and optical loss of the metal back contact, and **f** comparison of absorbance for perovskite solar cells with and without integrated moth eye texture

Moth eye textures have been intensively used to improve the light incoupling in biological or optoelectronic devices and systems [98–100]. Moth eye textures act like broadband anti-reflection coatings, so that the incoupling of light can be distinctly improved and the reflection is reduced [101, 102]. Figure 12a–d shows scanning electron microscopy (SEM) and atomic force microscope (AFM) images of moth eye surface textures [101]. The polymeric moth eye surface textures were fabricated by casting a Polydimethylsiloxane (PDMS) film on a crystalline silicon master, which was patterned by silicon semiconductor processing. The moth eye texture is characterized by periodically distributed nipples with a circular base arranged in a hexagonal grid. The surface profile of each nipple of the moth eye texture exhibits a paraboloid shape. It is shown in previous studies that paraboloid shaped nipple exhibit almost ideal incoupling properties [75, 103]. It is assumed that the moth eye texture used in this study has a diameter of 150 nm and a height of 200 nm [101] as shown in Fig. 12e, f. We have integrated the moth eye texture in a perovskite solar cell. Figure 13a shows a cross section of a textured solar cell. All interfaces of the solar cell structures are moth eye textured. The power density map for a wavelength of 750 nm is shown in Fig. 13b, and the calculated quantum efficiency is shown in Fig. 13e. Furthermore, the quantum efficiency of a flat or planar solar cell is included in Fig. 13e. For short wavelengths, the increased quantum efficiency is observed for the moth eye textured solar cell. For long wavelengths, the planar solar cell exhibits a higher quantum efficiency. The drop of the quantum efficiency is caused by the textured Al back contact. The Al back contact exhibits a high absorption, leading to high non-radiative optical losses for long wavelengths. This is confirmed by the power density map in Fig. 13b for 750 nm. The optics of the solar cell can be improved by using a planar Al reflector in combination with a textured interlayer. A schematic cross section of the solar cell is shown in Fig. 13c, and the corresponding power density map is shown in Fig. 13d. The optical loss of the back contact is distinctly reduced. A standing wave is formed in the solar cell. Hence it can be concluded that the moth eye texture leads to a good light incoupling, but no diffraction of the incident light is observed. The modified back contact design leads to a distinct gain in the quantum efficiency shown in Fig. 13e for long wavelengths. The total absorption of the solar cells and the absorption of the back contact are shown in Fig. 13f. The two solar cells with moth eye texture exhibit a total absorption close to unity. By minimizing the absorption losses of the contact layers the quantum efficiency of the solar cell can be increased. The flat solar cell exhibits a short-circuit current density of 21.4 mA cm^−2^, while the moth eye textured solar cells exhibit short-circuit current density of 22.6 and 23.1 mA cm^−2^. The moth eye textured Al reflector leads to an optical loss of 0.5 mA cm^−2^. In the following a guideline for realizing perovskite/silicon tandem solar cells with high short-circuit current density and energy conversion efficiency is provided.

### Perovskite/Silicon Tandem Solar Cell

**Fig. 14:**
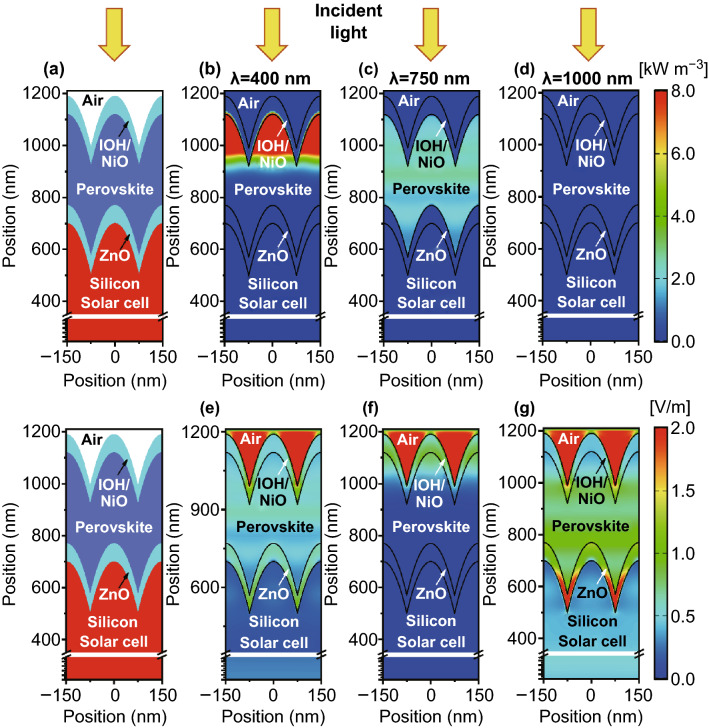
**a** Cross section of moth eye textured perovskite/silicon tandem solar cell. Power density map for incident wavelength of **b** 400 nm, **c** 750 nm, and **d** 1000 nm. Electric field distribution map for incident wavelength of **e** 400 nm, **f** 750 nm, and **g** 1000 nm

**Fig. 15:**
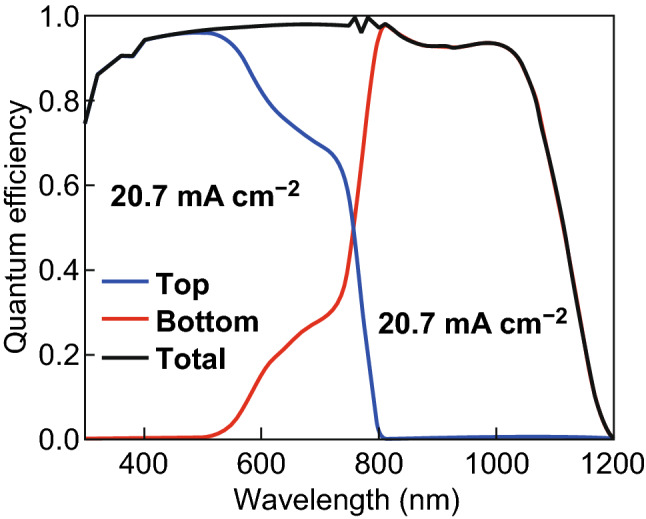
The calculated quantum efficiency of the top, bottom, and total perovskite/silicon solar cell under matched short-circuit current conditions

The perovskite and silicon absorbers are used as top and bottom solar cells. Crystalline silicon exhibits a bandgap of 1.15 eV so that the solar cell absorbs light up to almost 1200 nm. The upper theoretical limit of the short-circuit current density (Shockley-Queisser limit) is calculated as 46 mA cm^−2^. Experimentally realized single crystalline silicon solar cell exhibit short-circuit current density of 41–42 mA cm^−2^ and energy conversion efficiencies of ~ 26% [4]. This means that the maximal short-circuit current density of a perovskite/silicon tandem solar cell under current matching condition is 23 mA cm^−2^ [77]. Based on the best experimentally realized crystalline silicon solar cells it can be expected that the short-circuit current of the best perovskite/silicon tandem solar cells is in the range from 20 to 21 mA cm^−2^ [4, 37]. To determine an optical solar cell design, the optical wave propagation must be rigorously simulated. However, the thickness of the tandem solar cell is distinctly larger than the wavelength of the incident light, so that a rigorous simulation is computationally too complex [77]. Therefore, a hybrid approach is used to model wave propagation. The top cell consists of a double IOH/NiO front contact, the perovskite material, and a ZnO back contact. The silicon heterojunction bottom solar cell is described by an infinitely thick silicon substrate with a backside texture comparable to the front side texture of conventional single-junction crystalline silicon solar cells [4, 77]. To determine the quantum efficiency of the bottom solar cell, the light transmitted by the perovskite top solar cell in the bottom solar cell is calculated. Furthermore, it is assumed that light entering the bottom solar cell refracted by the textured back side of the silicon wafer. The quantum efficiency of the top and bottom solar cell is given by Eqs. 44 and 45, respectively

44 $${\text{QE}}_{\text{top}} \left( \lambda \right) \approx A_{\text{perovskite}} \left( \lambda \right)$$

45 $${\text{QE}}_{\text{bottom}} \left( \lambda \right) \approx QE_{\text{c} - \text{Si}} \left( \lambda \right) \times T_{\text{perovskite}} \left( \lambda \right)$$

where $A_{\text{perovskite}}$ and $T_{\text{perovskite}}$ are the absorption and transmission of the perovskite layer. ${\text{QE}}_{\text{c} - \text{Si}}$ is the quantum efficiency taken from literature for a silicon solar cell with record energy conversion efficiency. The schematic cross section of a perovskite/silicon tandem solar cell with integrated moth eye texture is depicted in Fig. 14a. The corresponding power density maps and electric field distributions under different wavelengths are shown in Fig. 14b–g. For 400 nm all the incident light is absorbed by the first 100 nm of the perovskite top cell. For a wavelength of 750 nm, most of the light is still absorbed by the top solar cell. Only a small fraction of the incident light enters the bottom cell. For an incident wavelength of 1000 nm almost, all light is transmitted in the bottom diode, where the light is absorbed. The calculated quantum efficiency for the top, bottom and total cells under short-circuit current matched condition are illustrated in Fig. 15. The short-circuit current density is matched for a perovskite layer thickness of the 350 nm. At approximately 770 nm, both top and bottom exhibit equal quantum efficiency of approximately 50%. The matched short-circuit current density reaches 20.7 mA cm^−2^ while the total short-circuit current density is 41.4 mA cm^−2^ which is very close to our predicted reference value taken from record efficiency silicon solar cell. The attained short-circuit current density is very close to the upper theoretical limit. To provide a realistic prediction of the energy conversion efficiency of the solar cells a description of the open-circuit voltage and the fill factor is required. The full understanding of the formation of the high open-circuit voltage is needed. It has been proposed that the high open-circuit voltage is caused by slow bulk recombination, low density of states in the conduction and valence band or low band tails [104–106].

In this study, we estimate an upper limit of the energy conversion efficiency by combining the optical simulation results with results for experimentally realized solar cells. The best experimentally realized silicon solar cells exhibit an open-circuit voltage of ~ 0.74 V and fill factor of 84.9%, while the best perovskite solar cells using a MAPbI_3_ absorber exhibits 1.182 V open-circuit voltage and 77% fill factor [37, 107]. The maximum energy conversion efficiency can be estimated at ~ 33%. By using a perovskite top solar cell with an optimum bandgap of 1.7 eV a further improvement of the open-circuit voltage and the energy conversion can be expected. If we assume that the same matching short-circuit current is achieved the final energy conversion efficiency will increase up to ~ 35%. We are currently in the process of investigating the optics of perovskite/silicon tandem solar cells with MAPbI_1−*x*_Br_*x*_ top cell absorbers. By increasing the bandgap of the top diode to 1.7 eV we like to approach energy conversion efficiencies of 35%.

## Summary

Fundamental energy conversion efficiency losses of solar cells have been identified by providing a review of thermodynamic and detailed balance limits. The Shockley-Queisser model provides a fundamental understanding of losses and limits of single and tandem solar cells. However, only recent work on more generalized detailed balance limits shows that the Shockley-Queisser model must be extended to take charge transport, e.g., non-radiative, into account. Furthermore, optical limits, e.g., imposed by the Yablonovitch limit, must be considered. It is shown that the optics of a solar cell has not only an influence on the short-circuit current but on all solar cell parameters. The influence of photon management on the solar cell parameters of a perovskite single-junction solar cell and a perovskite/silicon solar cell is discussed in greater details. Finite-difference Time-domain (FDTD) optical simulations are performed to investigate the single and tandem solar cells. The photon management of a perovskite single-junction solar cell can be predominately improved by an improved light incoupling and a reduction in optical losses in the solar cell. The photon management of silicon solar cell is more complex. In addition to improving light incoupling and minimizing optical losses, the light must be trapped in a solar cell. We have proposed a potential design for the perovskite/silicon tandem solar cells using a moth eye surface texture, which allows for an improving light incoupling. The proposed perovskite/silicon tandem solar cell exhibits an energy conversion efficiency of over 32% and a matched short-circuit current density of 20.7 mA cm^−2^.
